# Axisymmetric rotational stagnation point flow impinging radially a permeable stretching/shrinking surface in a nanofluid using Tiwari and Das model

**DOI:** 10.1038/srep40299

**Published:** 2017-01-12

**Authors:** Natalia C. Roşca, Ioan Pop

**Affiliations:** 1 Department of Mathematics, Faculty of Mathematics and Computer Science, Babeş-Bolyai University, 400084 Cluj-Napoca, Romania

## Abstract

In this paper, the problem of normal impingement rotational stagnation-point flow on a radially permeable stretching sheet in a viscous fluid, recently studied in a very interesting paper, is extended to a water-based nanofluid. A similarity transformation is used to reduce the system of governing nonlinear partial differential equations to a system of ordinary differential equations, which is then solved numerically using the function bvp4c from Matlab. It is found that dual (upper and lower branch) solutions exist for some values of the governing parameters. From the stability analysis, it is found that the upper branch solution is stable, while the lower branch solution is unstable. Sample velocity and temperature profiles along both solution branches are graphically presented.

The Navier-Stokes equations are the basic governing equations of fluid mechanics. This set of nonlinear partial differential equations has no general solution, and analytic solutions are rare. However, in certain flow problems similarity transformations may be possible, reducing the Navier-Stokes equations to a set of nonlinear ordinary differential equations which are much easier to solve. Similarity solutions not only describe fundamental physically significant problems but also serve as accuracy standards for full numerical solutions. Similarity transformations, which reduce the number of independent variables for partial differential equations, are possible only for problems with certain physical symmetries (Wang^1^). Since only the stretching transformations yield all significant solutions, one can use a simpler stretching method described by Hansen^2^ and Ames^3^. However, the existence of exact solutions is fundamental not only in their own right as solutions of particular flows, but also is important in accuracy checks for numerical solutions. In some simplified cases, such a rigid body travelling through a fluid (e.g., missile, sports ball, automobile, spaceflight vehicle), or in oil recovery industry (crude oil that can be extracted from an oil field is achieved by gas injection or equivalently), an external flow impinges on a stationary point called stagnation-point that is on the surface of a submerged body in a flow, for which the velocity at the surface of the submerged object is zero. A stagnation point flow develops and the streamline is perpendicular to the surface of the rigid body. The flow in the vicinity of this stagnation point is characterized by Navier-Stokes equations. By introducing coordinate variable transformation, the number of independent variables is reduced by one or more (Sin and Chio^4^). The classic problem of two-dimensional stagnation-point flows has been first analyzed exactly by Hiemenz^5^. The result is an exact solution for the flow directed perpendicular to an infinite flat plate. The axisymmetric stagnation flow towards a plate was solved by Homann^6^. Howarth^7^ and Davey^8^ extended the two-dimensional and axisymmetric flows to the three dimensional case, which is based on boundary layer approximation in the direction normal to the plane. We mention also here the paper by Naganthran *et al*.^9^ on the unsteady stagnation-point flow and heat transfer of a special third grade fluid past a permeable stretching/shrinking sheet. This class of solutions, describing the fluid flow near the stagnation region, exists on all solid bodies moving in a fluid. The stagnation region encounters the highest pressure, the highest heat transfer, and the highest rates of mass deposition.

Further it should be mentioned that Agrawal^10^ has discovered a new axisymmetric stagnation-point flow, obtaining also an exact solution of the Navier–Stokes equations. In contrast to the irrotational outer flow of Homann^6^, this flow is rotational in the far field. Agrawal^10^ derived his solution using spherical coordinates. Very recently, Weidman^11^ has extended Agrawal’s^10^ paper to the case of normal impingement of the rotational stagnation-point flow to a radially stretching sheet. Sample velocity profiles along both solution branches have been presented. A linear temporal stability analysis reveals that solutions along the upper branch are stable while those on the lower branch are unstable. Stretching/shrinking problems may have applications in polymer technology where one deals with stretching of plastic sheets and in metallurgy that involves the cooling of continuous strips (Fisher^12^). Goldstein^13^ has pointed out, that the new type of shrinking sheet flow is essentially a backward flow and it shows physical phenomena quite distinct from the stretching flow case. A very good collection of papers on stretching sheets can be found in the review paper by Wang^14^.

Manca *et al*.^15^ in an excellent review paper have shown that heat transfer can be enhanced by employing various techniques and methodologies, such as increasing either the heat transfer surface or the heat transfer coefficient between the fluid and the surface, that allow high heat transfer rates in a small volume. Cooling is one of the most important technical challenges facing many diverse industries, including microelectronics, transportation, solid-state lighting, and manufacturing. The addition of micrometer- or millimeter-sized solid metal or metal oxide particles to the base fluids shows an increment in the thermal conductivity of resultant fluids. Apart from the application in the field of heat transfer, nanofluids (nanometer particles in a fluid) can also be synthesized for unique magnetic, electrical, chemical, and biological applications. The novel concept of nanofluids, first introduced by Choi^16^ in 1995 has been proposed as a route to surpassing the performance of heat transfer fluids currently available. A very small amount of nanoparticles, when dispersed uniformly and suspended stably in base fluids, can provide impressive improvements in the thermal properties of base fluids. Nanofluids, which are a colloidal mixture of nanoparticles (1–100 nm) and a base liquid (nanoparticle fluid suspensions) describe the new class of nanotechnology-based heat transfer. It is worth mentioning that many references on nanofluids can be found in the books by Das *et al*.^17^, Nield and Bejan^18^, and Shenoy *et al*.^19^, and in the review papers by Buongiorno^20^, Buongiorno *et al*.^21^, Kakaç and Pramuanjaroenkij^22^, Wong and Leon^23^, Lee *et al*.^24^, Fan and Wang^25^, Mahian *et al*.^26^, Sheikholeslami and. Ganji^27^, etc.

The present paper is concerned with the extension of the paper by Weidman^11^ on the axisymmetric rotational stagnation point flow impinging on a radially stretching sheet in a water based cooper (Cu), alumina (Al_2_O_3_) and titanium (TiO_2_) nanofluids by using the mathematical nanofluid model proposed by Tiwari and Das^28^. In addition, we have also studied here the case of a shrinking sheet with a stability analysis for the multiple (dual) solutions. Therefore, we believe that the results are new and original, which can be used with great confidence by all those who are interested by stretching/shrinking sheet problems in nanofluids. It is worth mentioning to this end that Sohel *et al*.^29^ studied analytically the entropy generations in the circular shaped microchannel and minichannel using Cu and Al_2_O_3_ as the nanoparticle with H_2_O, ethylene glycol (EG) as the base fluids.

## Problem Formulation

**Figure 1:**
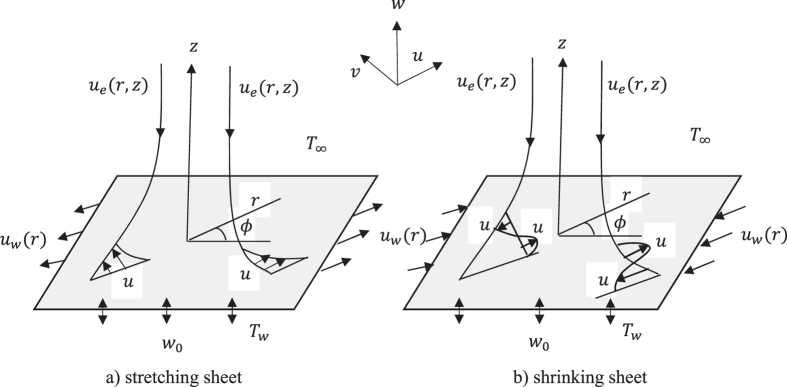
Geometry of the problem and coordinate system.

Consider the steady axisymmetric rotational stagnation point flow and heat transfer impinging radially a permeable stretching/shrinking surface in a water based nanofluid. The problem is formulated using cylindrical coordinates (*r, ϕ, z*), measured in the axial and radial directions, respectively, the flow being axisymmetric (∂/∂*ϕ* = 0) about the *z*– axis and also symmetric to the *z* = 0 plane. The stagnation line is at *z* = 0 and the domain of flow is in the upper half plane as shown in Fig. 1, where *u, v* and *w* are the velocity components along (*r, ϕ, z*)- axes. It is assumed that the velocity of the stretching/shrinking surface is *u*_*w*_(*r*), while that of the ambient fluid is *u*_*e*_(*r, z*) = 2*arz*, where *a* is a parameter, which measures the strength of the stagnation point flow having units (*LT*^−1^) (see Weidman^11^). It is also assumed that the constant temperature of the stretching/shrinking surface is *T*_*w*_, while the uniform temperature of the ambient fluid is *T*_∞_. Further, we assume that the mass flux velocity is *w* = *w*_0_, where *w*_0_ < 0 is for suction and *w*_0_ > 0 is for injection or withdrawal of the fluid.

Under these assumptions, the governing equations can be written in vectorial form as (see Tiwari and Das^28^)


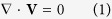



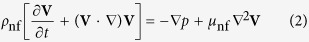



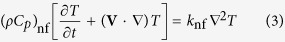


where **V** is the velocity vector, *T* is the fluid temperature, *p* is the pressure, *t* is the time, *μ*_nf_ is the dynamic viscosity of the nanofluid, *k*_nf_ is the thermal conductivity of the nanofluid and *ρ*_*nf*_ is the density of the nanofluid, which are given by the relations (4) and in Table 1 from the paper by Oztop and Abu Nada^30^


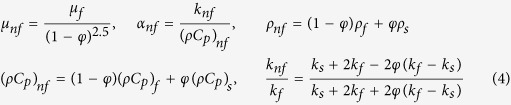


Here *α*_nf_ is the thermal diffusivity of the nanofluid, (*ρC*_*p*_)_*nf*_ is the heat capacity of the nanofluid and *φ* is the volume fraction of solid particle of the nanofluid.

Now, using the cylindrical coordinates (*r, ϕ, z*) and having in view that the flow is axisymmetric (∂*v*/∂*ϕ* = 0), Eqs (1, 2, 3) can be written as (see Bejan^31^):


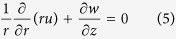



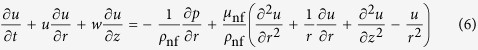



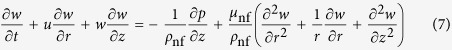



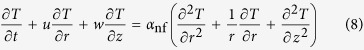


along with the initial and boundary conditions


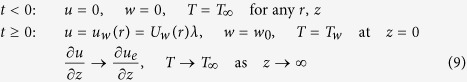


where 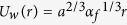 is the velocity of the stretching/shrinking surface, *α*_*f*_ is the thermal diffusivity of the base fluid and *λ* is the constant stretching/shrinking parameter with *λ* > 0 for the stretching surface and *λ* < 0 for the shrinking surface, respectively.

## Steady-State Case

We introduce for this case, the following similarity variables (Weidman^11^, and Kuznetsov and Nield^32^)


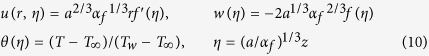


where prime denotes differentiation with respect to *η*. Using (10), Eq. (5) is satisfied automatically and since there is no longitudinal pressure gradient Eqs (6) and (8) reduce to the following ordinary differential equations


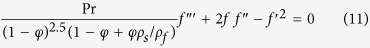



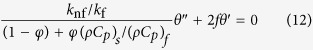


and the boundary conditions (9) become


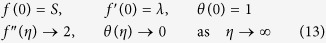


here 
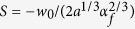
 is the dimensionless mass flux parameter, with *S* > 0 for suction and *S* < 0 for injection, respectively and Pr = *v*_*f*_/*α*_*f*_ is the Prandtl number where *v*_*f*_ is the kinematic viscosity of the base fluid.

The physical quantities of practical interest are the local skin friction coefficients *C*_*f*_ and the local Nusselt number *Nu*_*z*_, which are defined as


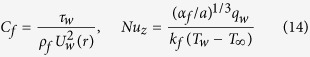


where *τ*_*w*_ is the skin friction or the shear stresses and *q*_*w*_ is the heat flux from the surface of the sheet, which are given by


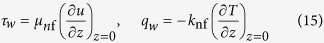


Substituting (10) into (15) and using (14), we obtain


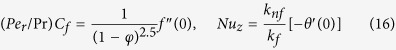


where 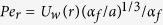 is the modified local Péclet number. In the case of *φ* = *S* = 0 and Pr = 1, the boundary-value problem for *f*(*η*) reduces to that of Weidman^11^.

## Stability Analysis

Merkin^33^, Weidman *et al*.^34^ and Roşca *et al*.^35^,^36^ have shown that the lower branch solutions are unstable (not physically realizable), while the upper branch solutions are stable (physically realizable). We test these features by considering Eqs (5, 6, 7, 8). Thus, we introduce the new dimensionless time variable 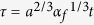. The use of *τ* is associated with an initial value problem and is consistent with the question of which solution will be obtained in practice (physically realizable). Using the variables *τ* and (10), we have


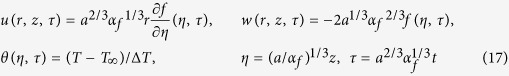


so that Eqs (5, 6, 7, 8) can be written as


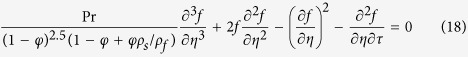



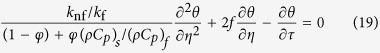


along with the boundary conditions (5) which become


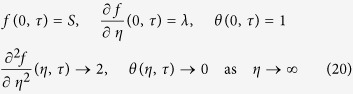


To determine the stability of the solution *f* = *f*_0_(*η*) and *θ* = *θ*_0_(*η*) satisfying the boundary-value problem (8)–(10), we write (see Merkin^33^; Weidman *et al*.^34^ and Roşca *et al*.^35^,^36^)


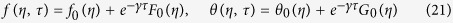


where *γ* is an unknown eigenvalue parameter, and *F*_0_(*η*) and *G*_0_(*η*) are small relative to *f*_0_(*η*) and *θ*_0_(*η*). Substituting (21) into Eqs (18) and (19) along with the boundary conditions (20), we have to solve the following linear eigenvalue problem


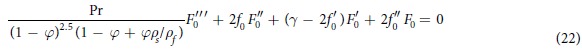



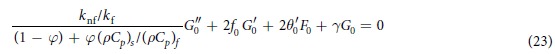


subject to the boundary conditions


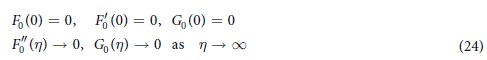


Solving the eigenvalue problem (22)–(24) we obtain an infinite number of eigenvalues *γ*_1_ < *γ*_2_ < *γ*_3_ < …. If the smallest eigenvalue is positive the flow is stable and if the smallest eigenvalue is negative the flow is unstable.

According to Harris *et al*.^37^, the set of possible eigenvalues can be determined by relaxing a boundary condition on *F*_0_(*η*) or *G*_0_(*η*). For the present problem, we relax the boundary condition 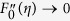 as *η* → ∞ and for a fixed value of *γ*, we solve the system of equations (22) and (23) subject to (24) along with the new boundary condition 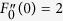.

## Results and Discussion

**Figure 2:**
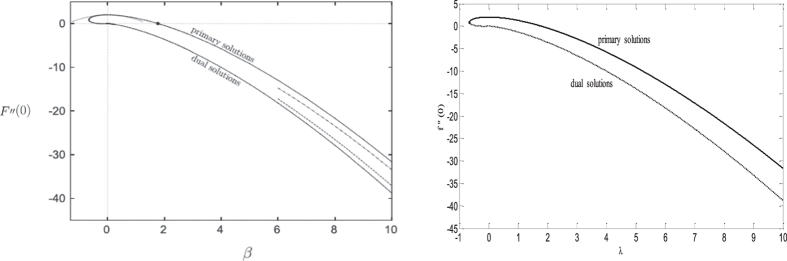
Comparisons of *f*″(0) when *φ* = 0, Pr = 1, *S* = 0 and *λ* = *β* (left Weidman^11^ and right the present results).

The ordinary differential equations (11) and (12), subject to the boundary conditions (13) have been solved numerically for several values of the suction *S*, volume fraction of the nanofluid *φ* and stretching/shrinking *λ* parameters when the Prandtl number Pr is fixed at Pr = 7, using the bvp4c function from Matlab software. The values of *φ* = 0, 0.1 and 0.2 have been taken as in the paper by Oztop and Abu-Nada^30^. The tutorial and examples of solving boundary value problems with bvp4c can be found in the book by Shampine *et al*.^38^. It is expected that the present problem may have more than one solution, therefore a good initial guess is needed to obtain the desired solutions of the ODEs (11) and (12). The asymptotic boundary conditions in (13) at *η* → ∞ are replaced by those at a large but finite value of *η* where no considerable variation in velocity, temperature, etc. occur as is usually the standard practice in the boundary layer analysis. In order to validate the obtained results we have compared in Fig. 2, the reduced skin friction coefficient *f* ″(0) when *φ* = 0, Pr = 1, *S* = *0* and *λ* = *β* (left Weidman^11^ and right the present results). It can be seen from this figure a favorable agreement with the above mentioned paper. Therefore we are confident that the present results are accurate and corect.

**Figure 3:**
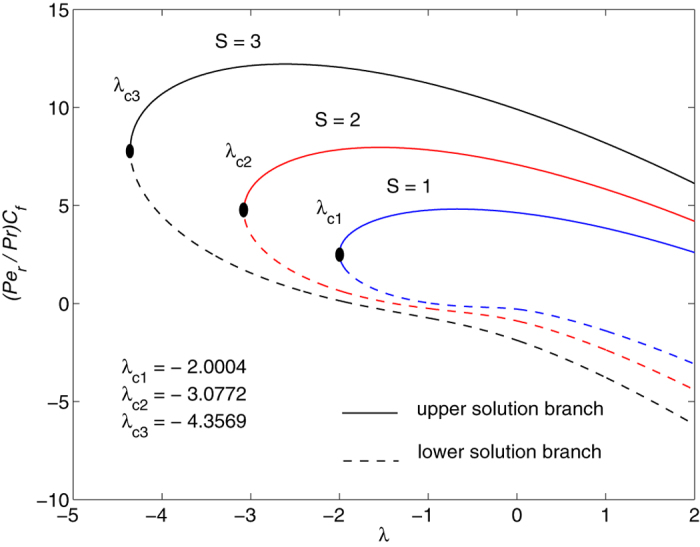
Variation of the skin friction coefficient as a function of *λ* for several values of *S* when *φ* = 0.1 and Pr = 7.

**Figure 4:**
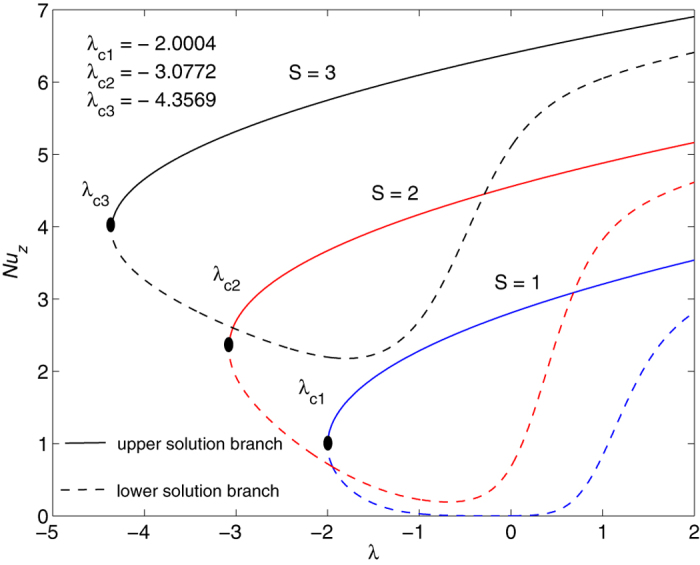
Variation of the local Nusselt number as a function of *λ* for several values of *S* when *φ* = 0.1 and Pr = 7.

**Figure 5:**
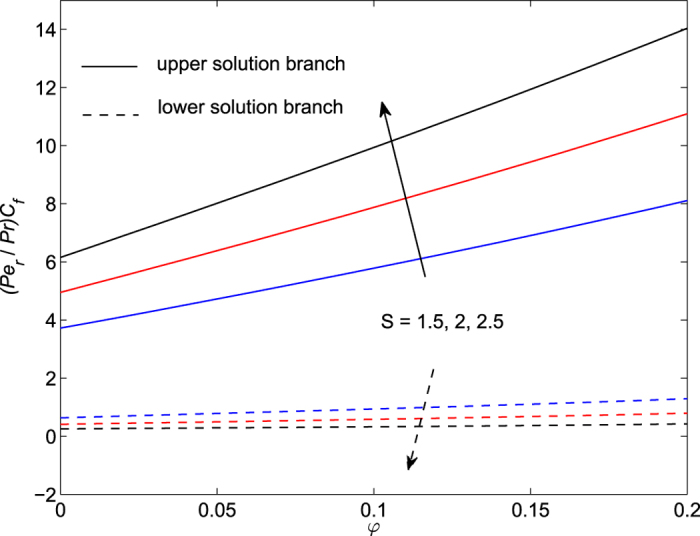
Variation of the skin friction coefficient as a function of *φ* for several values of *S* when *λ* = −1.95(<0) and Pr = 7.

**Figure 6:**
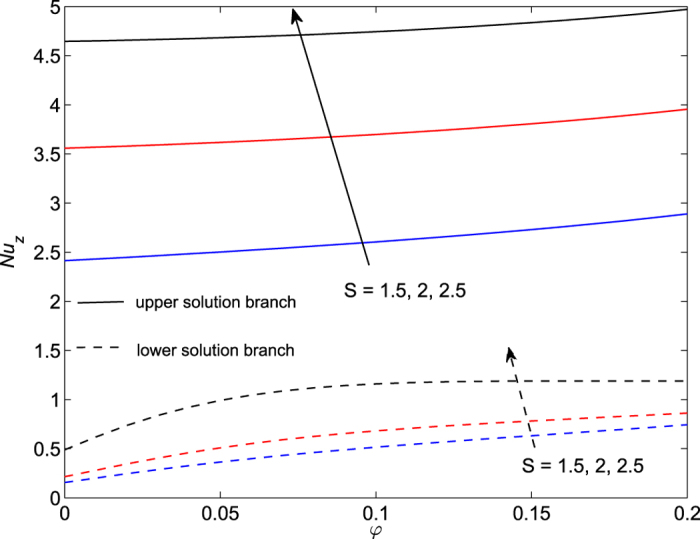
Variation of the local Nusselt number as a function of *φ* for several values of *S* when *λ* = −1.95(<0) and Pr = 7.

**Figure 7:**
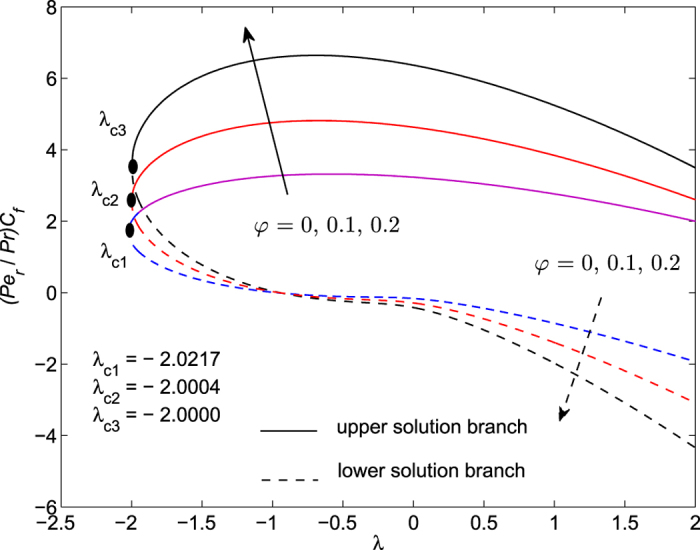
Variation of the skin friction coefficient as a function of *λ* for several values of *φ* when *S* = *1* and Pr = 7.

**Figure 8:**
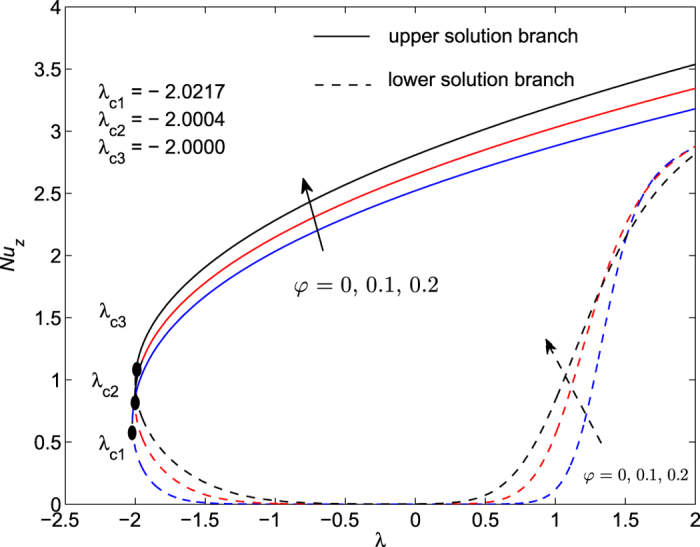
Variation of the local Nusselt number as a function of *λ* for several values of *φ* when *S* = *1* and Pr = 7.

**Figure 9:**
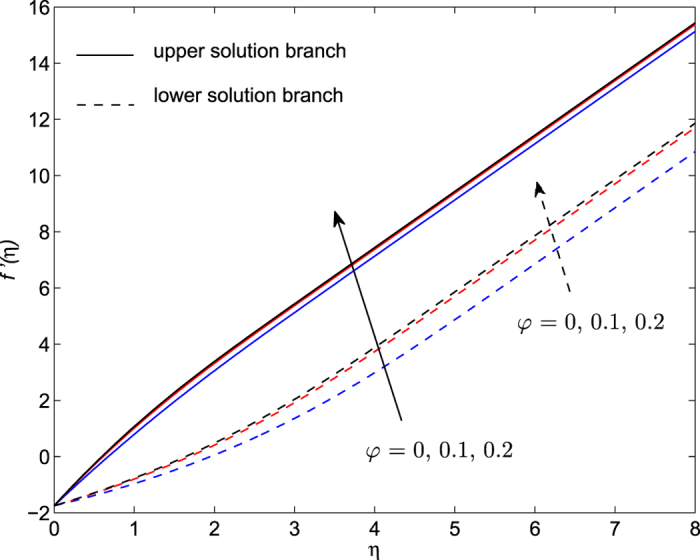
Dimensionless velocity profiles *f*′(*η*) for several values of *φ* when *S* = 1, Pr = 7 and *λ* = −1.75.

**Figure 10:**
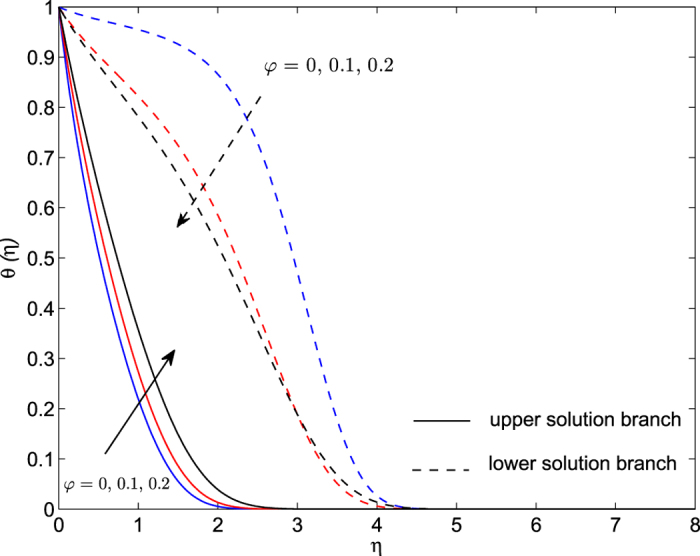
Dimensionless temperature profiles *θ*(*η*) for several values of *φ* when *S* = 1, Pr = 7 and *λ* = −1.75.

The obtained numerical results are displayed in terms of the skin-friction coefficient (*Pe*_*r*_/Pr)*C*_*f*_, local Nusselt number *Nu*_*z*_, dimensionless velocity *f* ′(*η*) and temperature *θ*(*η*) profiles, for different values of the parameters *S, λ and φ* with Pr = 7 (water). These results are shown in Figs 3, 4, 5, 6, 7, 8, 9, 10. We observe from Figs 3, 4, 5, 6, 7, 8 that the system of equations (11) and (12) subject to the boundary conditions (13) admits multiple (double) solutions, i.e. one upper branch solution and one lower branch solution, respectively. In order to ascertain which of the double solutions is expected to be stable, we find the eigenvalues *γ* in (21) by solving numerically Eqs (22) and (23) subject to the boundary condition (24) using the bvp4c routine. From the performed stability analysis it follows that the upper branch solutions are stable and physically realizable, while the lower branch solutions are not stable and hence physically not realizable. The smallest eigenvalues *γ* for several values of *φ* and *φ* when *S* = 1, Pr = 7 (water) are given in Table 2. It is worth mentioning that the smallest eigenvalues *γ* are positive for the upper branch solutions while the lower branch solutions have negative values of *γ*, which correspond to initial growth of disturbances, hence the flow is unstable. Although only the upper branch solutions are stable, the lower branch solution is also of mathematical interest as the boundary value problem (11)-(13) is concerned.

Figures 3, 4, 5, 6 are plotted to show the variation of the skin-friction coefficient (*Pe*_*r*_/Pr)*C*_*f*_ and local Nusselt number *Nu*_z_ with the stretching/shrinking parameter *λ* for several values of *S* and *φ* when Pr = 7.

The numerical computations shown in Figs 3 and 4 for (*Pe*_*r*_/Pr)*C*_*f*_ and *Nu*_*z*_ suggest that for these values of *φ* and Pr, the dual solutions exist for both stretching and shrinking sheets when *S* = 1: *λ* ≥ *λ*_*c*1_ = −2.0004; *S* = 2: *λ*≥*λ*_*c*2_ = −3.0772 *S* = 3: *λ* ≥ *λ*_*c*3_ = −4.3589. However, for*λ* < *λ*_*c*1_, *λ* < *λ*_*c*2_ and *λ* < *λ*_*c*3_ when *S* = 1, 2 and 3, respectively, solutions of Eqs (11) and (12) subject to the boundary conditions (13) do not exist and the full Navier-Stokes and energy equations should be solved. It is also seen from these figures that for the upper and lower branch solutions, both skin-friction coefficient and local Nusselt number increase with the suction parameter *S* as the shrinking parameter *λ*(<0) is fixed. Here *λ*_*ci*_(<0), *i* = 1, 2, 3 are the critical values of *λ*(<0) starting from which the boundary value problem (11)–(13) has at least one solution.

Figures 5 and 6 are plotted in order to present the variation of (*Pe*_*r*_/Pr)*C*_*f*_ and *Nu*_*z*_ with *λ* for several values of nanoparticle volume fraction *φ* when *S* = 1 and Pr = 7. It can be seen from these figures that dual solutions exist when *φ* = 0: *λ* ≥ *λ*_*c*1_ = −2.0217; *φ* = 0.1: *λ* ≥ *λ*_*c*2_ = −2.0004; *φ* = 0.2: *λ* ≥ *λ*_*c*3_ = −2.0000. However, for *λ* < *λ*_*c*1_, *λ* < *λ*_*c*2_ and *λ* < *λ*_*c*3_ when *φ* = 0, 0.1 and 0.2, respectively, solutions of Eqs (11) and (12) subject to the boundary conditions (13) do not exist and the full Navier-Stokes and energy equations should be solved. We observe here an increase of both skin-friction coefficient and local Nusselt number with nanoparticle volume fraction *φ* for the upper branch solution when the shrinking parameter *λ*(<0) is fixed.

It is also seen from Figs 3 and 4 that |*λ*_*ci*_| increases with the increase of suction parameter *S*, while |*λ*_*ci*_| decreases very slowly with the increase of *φ*, as can be seen in Figs 5 and 6. The range of the dual solutions increases with *S* and decreases with *φ*, as the strength of |*λ*| increases.

As shown in Figs 4, 6 and 8 the local Nusselt number *Nu*_*z*_ is always positive, i.e. the heat is transferred from the hot surface of the stretching/shrinking sheet to the cold fluid, which is consistent from a physical point of view. However, the fact that *Nu*_*z*_ increases with |*λ*| indicates that the stretching enhances the heat transfer at the surface, but the shrinking inhibits the effect of heat transfer at the surface.

From the numerical results shown in Figs 7 and 8 we observe that the skin-friction coefficient and the local Nusselt number for the upper branch solution increase with the suction parameter *S* when *φ* ∈ [0, 0.2] is fixed. We also see that heat flux at the surface increases for both upper and lower branch solutions (see Fig. 8).

The plots of the velocity *f* ′(*η*) and temperature *θ*(*η*) profiles with *η* for various values of *φ* when *S* = 1, Pr = 7 and *λ* = −1.75 are presented in Figs 9 and 10. The dual solutions are also obtained for both velocity and temperature distributions. It is seen that the lower branch solutions for these profiles exhibit a larger boundary layer thickness compared to the upper branch solutions. The enhancement of the value of nanofluid parameter *φ* increases the velocity profile for both upper and lower branch solutions, while it decreases the temperature profiles for lower branch solution and increases for upper branch solution. The fluid velocity is damped faster for a higher value of the parameter *φ*. It is noticed from Fig. 10 that the thermal boundary layer is blown away from the sheet with *φ* in the case of upper branch solution.

## Conclusions

This paper considered numerical solutions and stability analysis of the problem of normal impingement rotational stagnation-point flow on a radially permeable stretching/shrinking sheet in a nanofluid using the mathematical nanofluid model proposed by Tiwari and Das^28^. From this study, it results in the following important conclusions:

- Dual solutions exist for both stretching (*λ* ≥ 0) and shrinking cases (*λ*_*ci*_ ≤ *λ*<0) with curves which bifurcate at the critical values *λ*_*ci*_ < 0 of the governing ordinary (similarity) equations (11) and (12) subject to the boundary conditions (13).
- A stability analysis has been done to show that the first solution (upper branch) is stable, whereas the second solution (lower branch) is unstable.
- Skin friction coefficient (*Pe*_*r*_/Pr)*C*_*f*_ and the local Nusselt number *Nu*_*z*_ from the surface of the sheet is increased as the rate of suction is increased.
- The analysis of the present investigation plays a predominant role in the applications of science and technology. Particularly, the results of the present problem are of great interest in controlled metal welding or magnetically controlled coating of metals, in fusion engineering problems, polymer engineering, metallurgy, etc.

## Additional Information

**How to cite this article**: Roşca, N. C. and Pop, I. Axisymmetric rotational stagnation point flow impinging radially a permeable stretching/shrinking surface in a nanofluid using Tiwari and Das model. *Sci. Rep.* **7**, 40299; doi: 10.1038/srep40299 (2017).

**Publisher's note:** Springer Nature remains neutral with regard to jurisdictional claims in published maps and institutional affiliations.

## Figures and Tables

**Table 1:** Physical properties of fluid and nanoparticles (Oztop and Abu-Nada^30^).

Property		Water	Cu	Al_2_O_3_	TiO_2_
*c*_*p*_	(J/kg K)	4179	385	765	686.2
*ρ*	(kg/m^3^)	997.1	8933	3970	4250
*k*	(W/m K)	0.613	400	40	8.9538

**Table 2:** Smallest eigenvalues *γ* for several values of *φ* and *λ* when *S* = *1*, Pr = 7 (water).

*φ*	*λ*	*γ*	
		Upper branch	Lower branch
0.05	1	5.7226	–1.9896
	–1	3.5125	–1.9237
	–1.5	2.5078	–1.7722
	–1.9	1.1226	–0.9798
	–2	0.2245	–0.2185
0.1	1	5.6122	–1.9841
	–1	3.4547	–1.9130
	–1.5	2.4662	–1.7572
	–1.9	1.0889	–0.9559
	–2	0.0704	–0.0698
0.2	1	5.5457	–1.9818
	–1	3.4227	–1.9079
	–1.5	2.4459	–1.7508
	–1.9	1.0800	–0.9502
	–2	0.0245	–0.0245
